# Observed versus predicted mortality after isolated tricuspid valve surgery

**DOI:** 10.1111/jocs.16483

**Published:** 2022-04-06

**Authors:** Marco Russo, Guglielmo Saitto, Antonio Lio, Michele Di Mauro, Paolo Berretta, Maurizio Taramasso, Roberto Scrofani, Alessandro Della Corte, Sandro Sponga, Ernesto Greco, Matteo Saccocci, Antonio Calafiore, Giacomo Bianchi, Andrea Biondi, Irene Binaco, Ester Della Ratta, Ugolino Livi, Paul Werner, Carlo De Vincentiis, Federico Ranocchi, Marco Di Eusanio, Alfred Kocher, Carlo Antona, Fabio Miraldi, Giovanni Troise, Marco Solinas, Francesco Maisano, Guenther Laufer, Francesco Musumeci, Martin Andreas

**Affiliations:** ^1^ Department of Cardiac Surgery Medical University of Vienna Vienna Austria; ^2^ Department of Cardiac Surgery and Heart Transplantation San Camillo Forlanini Hospital Rome Italy; ^3^ Department of Cardiac Surgery IRCSS Policlinico San Donato Milan Italy; ^4^ Cardio‐Thoracic Surgery Unit, Heart and Vascular Centre, Maastricht University Medical Centre (MUMC) Cardiovascular Research Institute Maastricht (CARIM) Maastricht The Netherlands; ^5^ Cardiac Surgery Unit, Lancisi Cardiovascular Center Polytechnic University of Marche Ancona Italy; ^6^ Department of Cardiac Surgery University Heart Center of Zurich Zurich Switzerland; ^7^ Cardiac Surgery Unit Ospedale Fatenefratelli Sacco Milano Italy; ^8^ Department of Translational Medical Sciences, Unit of Cardiac Surgery, V Monaldi Hospital University of Campania "L. Vanvitelli" Campania Italy; ^9^ Cardiac Surgery Unit University Hospital of Udine Udine Italy; ^10^ Department of Cardiovascular, Respiratory, Nephrological, Anesthesiological, and Geriatric Sciences Sapienza University Rome Italy; ^11^ Cardiac Surgery Unit Poliambulanza Foundation Hospital Brescia Italy; ^12^ Prince Sultan Cardiac Center Ryiadh Saudi Arabia; ^13^ Pasquinucci Heart Hospital G. Monasterio Foundation Massa Italy

**Keywords:** valve repair/replacement

## Abstract

**Background:**

Aim of this study is to analyse the performances of Clinical Risk Score (CRS) and European System for Cardiac Operative Risk Evaluation (EuroSCORE)‐II in isolated tricuspid surgery.

**Methods:**

Three hundred and eighty‐three patients (54 ± 16 year; 54% female) were enrolled. Receiver operating characteristic analysis was performed to evaluate the relationship between the true positive fraction of test results and the false‐positive fraction for a procedure.

**Results:**

Considering the 30‐day mortality the area under the curve was 0.6 (95% confidence interval [CI] 0.50–0.72) for EuroSCORE II and 0.7 (95% CI 0.56–0.84) for CRS‐score. The ratio of expected/observed mortality showed underestimation when considering EuroSCORE‐II (min. 0.46–max. 0.6). At multivariate analysis, the CRS score (*p* = .005) was predictor of late cardiac death.

**Conclusion:**

We suggest using both scores to obtain a range of expected mortality. CRS to speculate on late survival.

## 1 INTRODUCTION

Isolated tricuspid valve (TV) surgery is a rarely performed procedure generally associated with a high incidence of postoperative adverse events and elevated mortality. A recently published analysis of 1041 patients treated in the United States showed in a 10‐year period a progressive increase in the number of operations performed per year. The overall operative mortality reported was 8.8% with a significant advantage of repair over replacement (*p* = .009).^1^

The preoperative risk assessment represents a key step in patient management. The European System for Cardiac Operative Risk Evaluation (EuroSCORE) II^2^ and The Society of Thoracic Surgeons (STS) score^3^ are currently used in clinical practice for preoperative risk estimation, both in surgical and transcatheter valve patients.^4^

These scoring systems are based on several different preoperative comorbidities. Despite they are not validated in the field of isolated tricuspid valve procedures. In this setting, several factors not contemplated by conventional scoring systems such as right ventricular function, etiology of disease, or liver function could play a special role in the patient's outcome. With the increasing number of transcatheter procedures for the treatment tricuspid insufficiency, a dedicated method to preoperatively address the risk profile of a patient is of increasing importance.^5^, ^6^

Indeed, just recently, based on the results of a multivariable model on more than 2000 patients, an easily clinical calculable score (Clinical Risk Score [CRS] score) was established by LaPar and co‐authors to estimate the probability of perioperative mortality and major morbidity after isolated TV surgery.^7^ Using CRS values derived from rounded adjusted odds ratios for each factor entered into the final regression models, probability event rates were calculated for categories of clinical risk scores with a CRS score range from 1 to 10+. Depending on the calculated total CRS, predicted probability of death ranged from 2% to 34%. However, the CRS score is neither externally validated nor largely used in the current clinical practice.

The aim of the current study is the comparison the performances of two different scoring systems, EuroSCORE II and CRS score, in the setting of isolated tricuspid valve surgery.

## 2 MATERIALS AND METHODS

### 2.1 Registry design and data collection

The International SUR‐TRI Registry is a multicenter registry initiated by the Department of Cardiac Surgery at the Medical University of Vienna and involving 12 international cardiac surgery units with experience in the surgical management of isolated tricuspid disease. The registry is not supported by external funding. The study was approved by the Ethical Committee of the Medical University of Vienna (1289/2019) and at each center according to local indication.

All adults patients (age > 18) operated over a 10 years period (2008–2019) in the participating centers were enrolled in the registry. Only patients undergoing isolated tricuspid valve repair or replacement procedure were included and any concomitant valve surgery, coronary surgery, atrial fibrillation surgery, congenital lesions surgery were excluded. Criteria for performing repair over replacement technique were not standardized by a study protocol but were associated with local practice and surgeon's decision.

For each patient included in the study, the baseline preprocedural clinical features, intraoperative characteristics and results at 30 days and at follow‐up were collected retrospectively. Long‐term follow‐up was performed by institutional database analysis or direct assessment by local investigators through study visits. Follow‐up was 98% complete with a mean duration of 40 months (range: 1–122 months). Informed consent was obtained according to local regulation. All methods were carried out in accordance with current guidelines and regulations.

### 2.2 Statistical analysis

Descriptive statistical methods were applied to depict the study population at baseline. Continuous normally distributed variables are presented as means ± standard deviation; skewed data as median and interquartile range (25th and 75th percentile). Categorical variables are presented as numbers (%). Differences between groups are compared with the Student's *t* test for normally distributed variables and the Mann–Whitney *U* test for not normally distributed ones. Categorical variables are summarized as the number and percentage of subjects in each category and differences compared with the Pearson‐*χ* ^2^ test.

Thirty‐day mortality is defined as the rate of death that occurred up to the 30th postoperative day after the surgical procedure. In‐hospital mortality is defined as any death which occurred before discharge from the hospital at any time interval while operative mortality is defined as any death which occurred before discharge or up to the 30th postoperative day when the patient could have left alive the hospital before (30‐day mortality + in hospital mortality). In our study population, in‐hospital and operative mortality were equal.

All deaths for unknown reasons were considered cardiac death for statistical purposes. The observed mortalities are described as a linear rate (%).

The expected‐to‐observed mortality ratio were obtained by dividing the expected number of events according to the risk score by the observed one, with a value of 1.0 indicating optimal prediction, value < 1.0 an underestimation of the risk score while value >1.0 an overestimating effect.

Receiver operating characteristic (ROC) curves were generated for the risk scoring systems analyzed (EuroSCORE II and CRS score). The ROC curves are used in clinical research to determine a special cut‐off value for a special test or, in our study, a special calculator. This algorithm analyses the relationship between the true positive fraction of test results and the false‐positive fraction for a procedure. To measure the accuracy of the risk calculator, the area under the curve (AUC) is reported. It could vary between 0.5 (lowest accuracy) and 1.0 (highest accuracy). Results are presented as AUC and 95% confidence intervals (CI). Kaplan–Meier analysis was performed to assess freedom from cardiac death.

The Cox proportional hazards model was used to evaluate the influence of variables on late cardiac survival. Sixteen preoperative variables, including age, male sex, endocarditis, New York Heart Association (NYHA) Class III/IV, peripheral vascular disease, previous stroke, chronic obstructive pulmonary disease (COPD), diabetes, hypertension, left ventricular ejection fraction (LVEF), urgent or emergent surgery, preoperative EuroSCORE II and CRS score, previous cardiac surgery, repair or replacement technique, CPB time, and beating heart technique were included in the univariate analysis for the prediction of late cardiac survival. Among them, risk factors with a *p* value less than .1 were included in the multivariate analysis model.

This study represents an “as‐treated analysis”; given the retrospective design of the study, data regarding the “intention‐to‐treat population,” and the relative cross‐over from one treated to the other one were not collected. Statistical analysis has been executed with the IBM SPSS Statistics 27.0 (IBM Corp).

### 2.3 Risk score calculation

Risk score were recorded from the analysis of the medical documentation when specifically stated in the operation report of the patient or retrospectively recalculated. The online calculator (http://www.euroscore.org/calc.html) was used for EuroSCORE II calculations. The CRS score was calculated as published by LaPar et al.^7^ All variables were calculated and classified according to the exact definitions set out in each of the scoring systems.

## 3 RESULTS

### 3.1 Patient demographic

A total of 383 consecutive patients (mean age 54 ± 16 years old; 54% female) were enrolled in the International SUR‐TRI Registry. Indication for surgery was endocarditis in 23%, functional regurgitation in 45%, rheumatic disease in 10%, and other etiologies (degenerative, pacemaker‐related, and carcinoid syndrome) in the remaining 22%. In 20% of cases, surgery was executed in an urgency/emergency setting. 157 (40%) patients had already undergone left side cardiac surgery and severe symptoms (NYHA III/IV) were present in 47% of the population. Preoperative left ventricular ejection fraction was 56 ± 10%. Moderato‐severe tricuspid regurgitation (TR) was present in 96% of the population while in the rest indication was associated with valve stenosis or severe mixed disease. An isolated repair procedure (TVr) was performed in 48% of case (*n* = 185) and a beating heart strategy was applied in 38% of the whole population (*n* = 149). In the TVr group (*n* = 185), 68% underwent tricuspid ring implantation, 15% suture annuloplasty, 10% tricuspid valve bicuspidalization while remaing cases underwent other techniques as clover technique, vegetation removal, pericardial patch augmentation. In the TVR group (*n* = 198) 76% received a biological valve, 12% a mechanical valve while in 12% this data was unknown. A right mini‐thoracotomy approach was performed in 21% of patients.

The median EuroSCORE‐II was 2.93% (quartiles 1.50–6). The median CRS mortality score was 4 (quartiles 2–6) with a median predicted mortality of 6%.

### 3.2 In‐hospital mortality

Twenty‐four patients experienced in‐hospital death (6.26%). Twelve (3.1%) deaths were defined as cardiac, 7 (1.8%) as sepsis‐related, 3 (0.8%) respiratory, 1 (0.26%) as multiorgan failure, and 1 (0.26%) as liver dysfunction.

The expected/observed in‐hospital mortality ratio was 0.46 for EuroSCORE II and 0.95 for CRS score.

**Figure 1:**
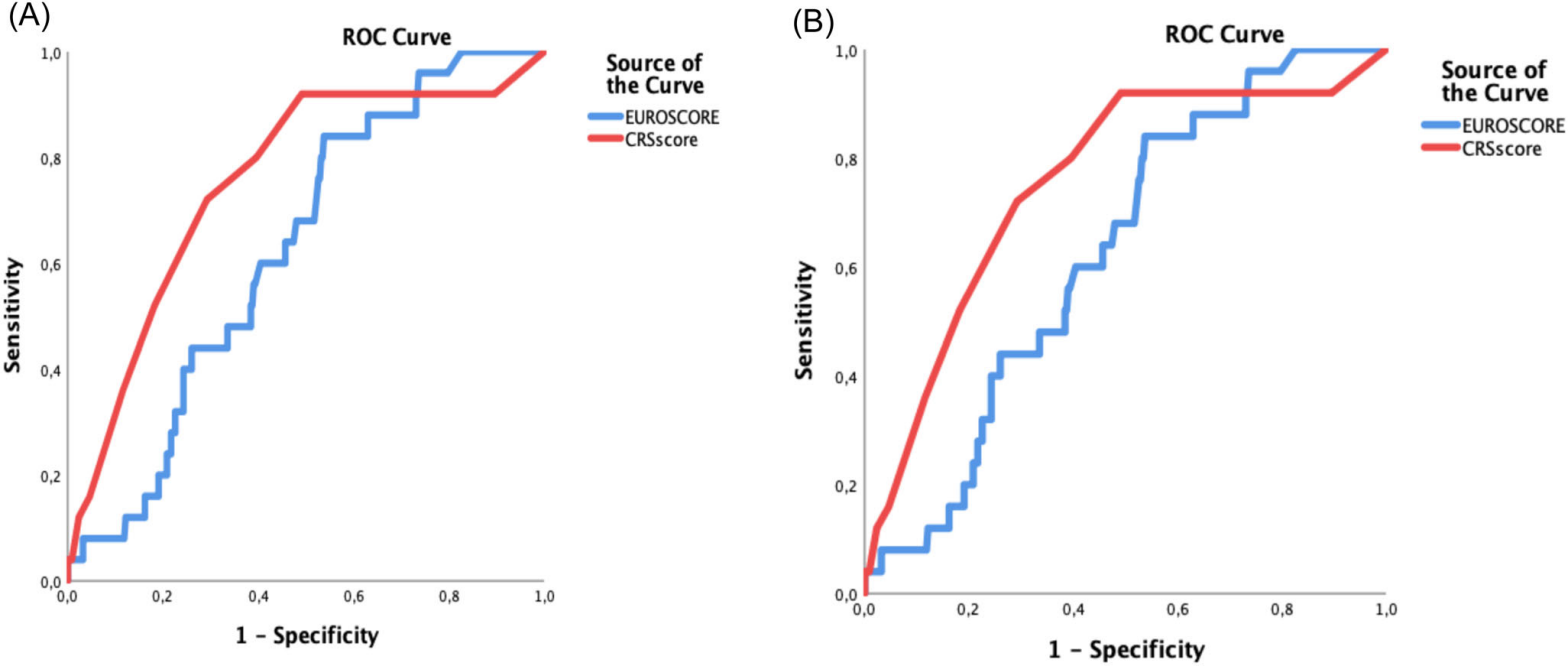
(A) Receiver‐operating characteristic (ROC) curves for all patients (*n* = 383) considering in‐hospital mortality. CRS score (red line) and EuroSCORE II (blue line). (B). ROC curves for all patients (*n* = 383) considering 30‐day mortality. CRS score (red line) and EuroSCORE II (blue line). CRS, Clinical Risk Score; EuroSCORE II, European System for Cardiac Operative Risk Evaluation II

For the whole patient population (*n* = 383), the AUC for the EuroSCORE II was 0.63 (95% CI 0.53–0.73) and 0.74 (95% CI 0.63–0.85) for CRS score (Figure 1A).

### 3.3 Thirty‐day mortality

Thirty‐day mortality rate was 4.96% (*n* = 19). 11 patients experienced a cardiac death (57%), 5 (26%) a sepsis‐related death, and 2 (10%) pulmonary while 1 (5%) associated with multiorgan failure.

The expected/observed 30‐day mortality ratio was 0.62 for EuroSCORE II and 1.2 for CRS score. When considering the 30‐day mortality (*n* = 383) the area under the curve (AUC) was calculated as 0.60 (95% CI 0.50–0.72) for the EuroSCORE II and 0.70 (95% CI 0.56–0.84) for CRS score (Figure 1B).

#### 3.3.1 Repair versus replacement

A subgroup analysis was performed to compare results of repair (TVr; *n* = 187) versus replacement (TVR; *n* = 196) approaches.

Patients in the TVr group were older (57 ± 16 vs. 53 ± 16 years, *p* = .019), less symptomatic (NYHA Class III/IV 42% vs. 51%, *p* = .06) and were less frequently previously operated on (32% vs. 48%, *p* = .001). Table 1 resumes the main pre‐ and intraoperative demographic features.

**Table 1:** Baseline patient characteristics

Variable	All patient (*n* = 383)	Repair (*n* = 187)	Replacement (*n* = 196)	*p*
Age, *n* ± *SD*	54 ± 16	57 ± 16	53 ± 16	**.01**
Female sex, *n* (%)	208 (54)	100 (54)	108 (54)	.9
BMI, kg/m^2^ ± *SD*	24.8 ± 6.9	24.6 ± 6.3	24.8 ± 7.1	.4
Diabetes, *n* (%)	43 (14)	27 (14)	26 (13)	.14
NYHA III–IV, *n* (%)	179 (47)	78 (42)	101 (51)	.06
Previous stroke, *n* (%)	20 (5)	9 (5)	11 (5)	.75
Dialysis, *n* (%)	12 (3)	4 (2)	8 (4)	.28
Ejection fraction, %	56 ± 10	57 ± 9	56 ± 10	.1
Moderato/severe TR, *n* (%)	346 (94)	179 (93)	167 (95)	.1
Previous cardiac surgery, *n* (%)	157 (41)	61 (32)	96 (48)	**.003**
Endocarditis, *n* (%)	90 (23)	31 (18)	59 (29)	**.002**
GFR, ml/min ±*SD*	76 ± 36	75 ± 32	77 ± 40	.1
Urgency/emergency, *n* (%)	78 (20)	34 (18)	44 (22)	.35
Median sternotomy, *n* (%)	317 (82)	160 (86)	157 (79)	.5
Ring annuloplasty, *n* (%)	129 (33)	129 (68)	‐	–
Beating heart, *n* (%)	149 (38)	59 (31)	90 (45)	**.006**
Cross‐clamp, min ± *SD*	59 ± 34	59 ± 33	59 ± 35	.9
CPB time, min ± *SD*	97 ± 52	90 ± 48	103 ± 56	**.02**

Abbreviations: CPB, cardio‐pulmonary bypass; GFR, glomerula filtration rate; NYHA, New York Heart Association; *SD*, standard deviation; TR, tricuspid regurgitation.

Thirty‐day mortality was 3.2% in TVr (*n* = 6) vs 6.6% in TVR (*n* = 13) (*p* = .1).

The expected/observed 30‐day mortality ratio for the EuroSCORE II in the TVr and TVR groups was 0.67 and 0.57, respectively (Table 2). Further, the expected/observed 30‐day mortality ratio for CRS score was 1.5 (TVr) and 0.90 (TVR).

**Table 2:** ROC analysis for 30‐day mortality.

Variable	All patient (*n* = 383)	Repair (*n* = 185)	Replacement (*n* = 198)
Observed 30 day mortality, *n* (%)	19 (5.19)	6 (3.2)	13 (6.6)
EuroSCORE II			
Median, quartiles %	2.93 (1.5–4,8)	2.2 (1.3–4.4)	3.8 (2.1–6.9)
Expected mortality, *n*	11.2 (2.9)	4.07 (2.2)	7.5 (3.8)
AUC	0.60	0.66	0.66
Expected/observed	0.60	0.67	0.57
CRS score			
Median, quartiles	4 (2–6)	3.5 (2–6)	4 (2–6)
Expected mortality, *n* (%)	22.9 (7)	9.25 (5)	11.8 (6)
AUC	0.70	0.76	0.75
Expected/observed	1.2	1.5	0.90

Abbreviations: AUC, area under the ROC curve; CRS, Clinical Risk Score; EuroSCORE II, European System for Cardiac Operative Risk Evaluation II; ROC, receiver operating characteristic.

**Figure 2:**
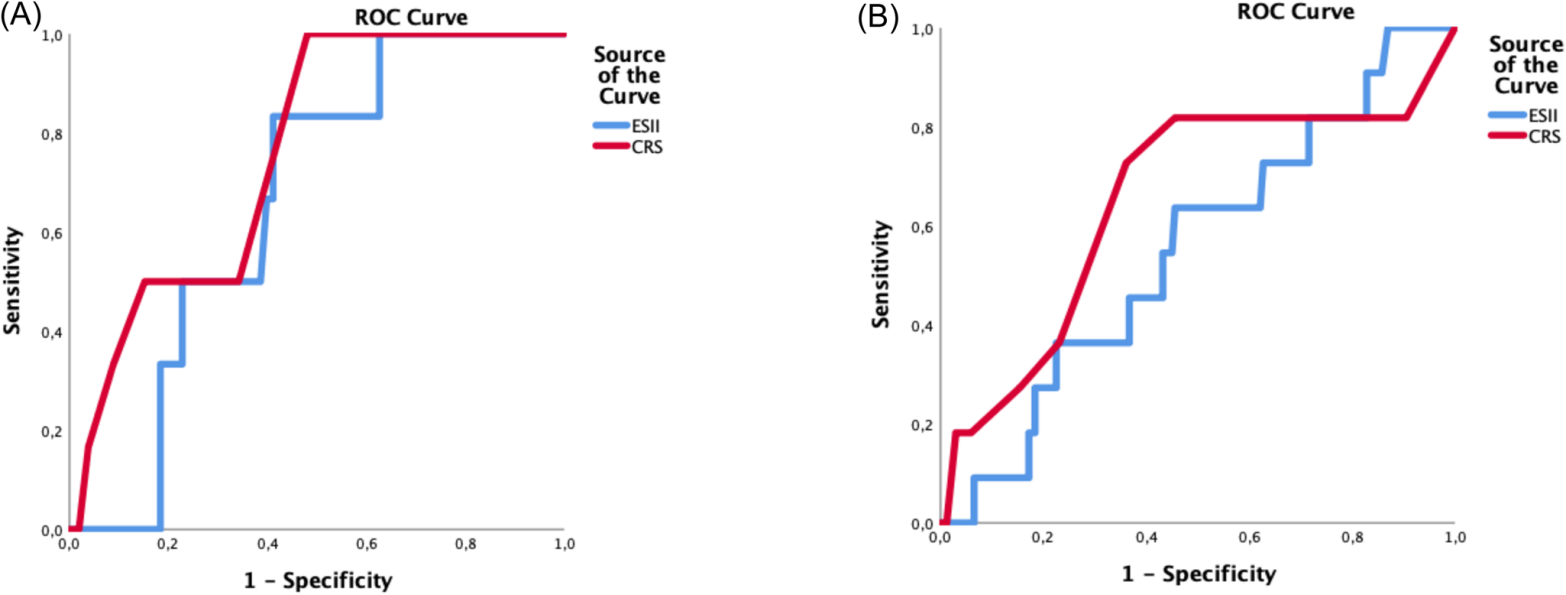
(A) Receiver‐operating characteristic (ROC) curves for patient treated with tricuspid valve repair (TVr; *n* = 185) for 30 day mortality. CRS score (red line) and EuroSCORE II (blue line). (B) ROC curves for patient treated with tricuspid valve replacement (TVR; *n* = 198) for 30 day mortality. CRS score (red line) and EuroSCORE II (blue line). CRS, Clinical Risk Score; EuroSCORE II, European System for Cardiac Operative Risk Evaluation II

In the TVr (*n* = 185) subgroup, AUCs calculated for 30‐day mortality were 0.66 (95% CI 0.52–0.80) and 0.76 (95% CI 0.61–0.91) for the EuroSCORE II and CRS score, respectively (Table 2) (Figure 2A). In the TVR group (*n* = 198), AUCs were 0.55 (95% CI 0.38–0.61) for EuroSCORE II and 0.65 (95% CI 0.46–0.83) for CRS Score respectively (Figure 2B).

Table 3 Describes the results of the ROC analysis for in‐hospital mortality.

**Table 3:** ROC analysis for in‐hospital mortality

Variable	All patient (*n* = 383)	Repair (*n* = 185)	Replacement (*n* = 198)
Observed In‐hospital mortality, n (%)	24 (6.26)	9 (4.8)	15 (7.6)
EuroSCORE II			
AUC	0.62	0.62	0.58
Expected/observed	0.46	0.45	0.50
CRS score			
AUC	0.74	0.79	0.70
Expected/observed	0.96	1.02	0.78

Abbreviations: AUC, area under the ROC curve; CRS, Clinical Risk Score; EuroSCORE II, European System for Cardiac Operative Risk Evaluation II; ROC, receiver operating characteristic.

### 3.4 Late results

During the follow‐up period a total of 54 patients did not survive due to cardiac reasons. Freedom from cardiac death was 85 ± 2%, 81 ± 2%, and 76 ± 3% at −3, −5, and −7 years, respectively. Univariate analysis showed that age, NYHA Class III/IV, TV replacement, LVEF < 55%, COPD, preoperative EuroSCORE II as well as CRS score, and urgent surgery were predictive factors for cardiac death during follow‐up. No association with type of valve implanted, biological versus mechanical, has been recorded. Table 4 presents the results of the univariate analysis.

**Table 4:** Univariate analysis for late cardiac survival.

	*p* value	OR	Lower CI	Upper CI
Age	.001	1.02	1.01	1.04
NYHA III/IV	.006	2.04	1.22	3.45
COPD	.02	2.48	1.41	4.37
Hypertension	.007	2.11	1.23	3.63
CRS score	<.001	1.24	1.14	1.35
EuroSCORE II	<.001	1.14	1.04	1.21
Urgent/emergency	<.001	2.75	1.69	4.49
LVEF < 50%	<.001	2.48	1.52	4.76
TV replacement	.08	1.52	0.95	2.45

Abbreviations: CI, confidence interval; COPD, chronic obstructive pulmonary disease; CRS, calculable risk score; LVEF, left ventricle ejection fraction; NYHA, New York Heart Association; TV, tricuspid valve.

At multivariate analysis, the CRS score (odds ratio [OR]: 1.14; 95% CI: 1.1–1.3; *p* = .005), LVEF < 55% (OR: 2.1; 95% CI: 1.04–3.5; *p* = .035), and urgent/emergent operation (OR: 1.9; 95% CI: 1.1–1.3; *p* = .014) were identified as predictors of late cardiac death (Table 5).

**Table 5:** Multivariate analysis for late cardiac survival

	*p* value	OR	Lower CI	Upper CI
CRS score	.005	1.14	1.04	1.21
Urgent/emergency	.035	1.93	1.04	3.5
LVEF < 50%	.014	2.14	1.17	3.91

Abbreviations: CI, confidence interval; CRS, calculable risk score; LVEF, left ventricle ejection fraction.

## 4 DISCUSSION

The clinical and prognostic importance of tricuspid valve disease has been increasingly studied in the past few years and the optimal treatment strategy, as well as the proper timing for invasive approaches, is still a matter of debate.^8^, ^9^ Although the tricuspid valve has been for a long time termed the “forgotten valve,” recently the rapid increase of recognition and the proper diagnosis of tricuspid disease have largely changed the attitude of the international cardiovascular community regarding this topic became the focus of intense research, debate and discussion.^10^

Isolated tricuspid operations have been historically associated with poor outcomes according to the presence of right ventricle dysfunction or systemic infection status associated with endocarditis. The outcomes reported in our series (30‐day mortality 4.9% (whole population), 3.2% in TVr vs. 7.6% in TVR) are in consistence with previously published data. Alqahtani and co‐authors reported a large cohort of 1364 patients describing 30‐day mortality of 8% and 10%, respectively, for repair and replacement techniques as well as an elevated incidence of perioperative adverse events such as stroke (2.3%), vascular complications (5.3%), acute renal failure needing dialysis (4.4%), cardiac tamponade (2.5%), and new pacemaker implantation (10.9%).^11^ Furthermore, the long‐term outcomes of tricuspid surgery have been reported by Saran et al.^12^ in a single‐center experience regarding more than 2000 patients of whom 9% (*n* = 221) isolated procedures (mean age 67 ± 13 years, 54% functional etiology of TR), with overall survival of 54%, 29%, and 13% at 5, 10, and 15 years, respectively. They demonstrated that replacement is associated with reduced long‐term survival. Our series confirmed a higher risk profile of patients undergoing TVR with an almost 1.5‐fold increased mortality rate (operative mortality was 4.8 [*n* = 9] vs. 7.8% [*n* = 15]).

The transcatheter approach for the treatment of patients affected by severe tricuspid regurgitation and judged to be at “high risk” for conventional surgery has been recently described with promising results.^13^, ^14^, ^15^ Data from the International Multisite Transcatheter Tricuspid Valve Therapies (TriValve) Registry reported the results of 312 consecutive cases (mean EuroSCORE II 9%) in 18 different centers worldwide with a 30‐day mortality of 3.6% and overall actuarial survival at 1.5 year of 77%.^16^, ^17^ Since transcatheter solutions for TR are rapidly increasing and several devices are under evaluation,^18^ a comprehensive preoperative assessment of the patients is mandatory to refer them either to surgery, interventional or conservative treatment. In this setting, the general statement “at high risk for surgery” based on the EuroSCORE II calculation or even the STS score is not related to evidence and both calculators are not specifically designed or validated for isolated tricuspid valve procedures.

Therefore, the CRS score has been developed to estimate perioperative mortality and morbidity of isolated tricuspid surgery based on a large analysis of operative outcomes of 50 North American centers. On basis of nine parameters (age, female sex, previous stroke, NYHA class, dialysis, COPD, LVEF, previous cardiac operation, and emergency surgery) obtained after a multiregression model analysis, a score value is obtained and associated with a percentage of expected mortality or morbidity. The internal validation of the statistical performance of the mortality and morbidity regression models was described with an AUC of 0.74 and 0.76 for mortality and composite morbidity after surgery.^7^ No data are so far reported regarding an external validation of the CRS score and its application in the current clinical practice is still not standardized.

In the present series we report the results of a multicenter experience of 383 consecutive patients enrolled in 12 different international centers with the aim to compare the performance of EuroSCORE II and CRS scores in predicting in‐hospital, 30‐day mortality, and late cardiac survival.

Herein we describe an AUC of 0.63 (EuroSCORE II) and 0.74 (CRS score), respectively, for the two scores considering in hospital mortality and AUCs of 0.60 and 0.70 for 30‐day mortality. These data confirm the previously mentioned internal validation of the CRS score.

When dealing with the ROC analysis we should consider that a model with an AUC of 0.50 represents a model with discrimination equal to chance alone while a test with a value of 1.0 has perfect discrimination between outcomes alternatives. Since no large data have been reported for tricuspid procedures, we can make a comparison with current scoring system applied to the more common executed aortic valve replacement. In a large series of 1066 patients affected by aortic valve stenosis and surgically treated, Wendt and co‐authors described an AUC of 0.75 for both the additive and logistic EuroSCORE I while the EuroSCORE II and the STS score had an AUC of 0.72.^19^ Similarly, Barili and co‐authors described an AUC of 0.81 for EuroSCORE I, 0.79 for EuroSCORE II, and 0.78 for ACEF score in a similar series of 1758 patients.^20^

The results of our study show that both the EuroSCORE II and the CRS score have an acceptable, but still not optimal, predictive value in the setting of isolated tricuspid surgery. More the CRS score seems to better predict mortality when compared with its counterpart. The AUCs reported are in the range of other risk calculator systems currently used in clinical practice. Since EuroSCORE II was originally designed to estimate 30‐day mortality and CRS was developed from results of in‐hospital mortality we analyzed the score's performances in different settings. Interestingly, we observed that EuroSCORE II is underestimating all mortality rates (ratio expected/observed min.0.46‐max.0.66) while the CRS score is slightly overestimating them (ratio 0.96–1.2) in the whole population, while it underestimated mortality in the TVR group. CRS showed very promising ratio values when dealing with in‐hospital mortality in the isolated repair group (1.02). This data could be associated with the vast majority (86%) of patients analyzed it the LaPar cohort (*n* = 2025) who were operated on isolated tricuspid reconstruction.

Interestingly, when considering late results, in the present analysis, CRS score (OR: 1.14; 95% CI: 1.1–1.3; *p* = .005), LVEF < 55% (OR: 2.1; 95% CI: 1.04–3.5; *p* = .035), and urgent/emergent operation (OR: 1.9; 95% CI: 1.1–1.3; *p* = .014) were identified as independent predictors of late cardiac death. According to our data CRS score but not EuroSCORE II may be used to speculate on late cardiac survival of this rare cohort of patients.

Wang et al.^21^ have recently reported the external validation of three different scoring systems, the EuroSCORE II, the MELD Score, and the CRS score in patients undergoing isolated tricuspid surgery. This study described a single‐center cohort of 207 patients enrolled at the Cleveland Clinic, USA. The AUCs were 0.83 (EuroSCORE II), 0.60 (CRS score), and 0.74 (MELD score). Furthermore, the calibration method showed similar power for EuroSCORE II and STS‐TVS, with a slight overestimation of mortality.

To the best of our knowledge, this is the first study that aimed to validate risk score for isolated tricuspid procedures in a multicenter design. The stratification of the patient profile and risk estimation are still lacking in the field of tricuspid disease. Despite this, the current era of clinical practice sees tricuspid disease as a hot topic both in international conferences and every day's heart team discussions. According to our data we believe that the combination of these two scores considering the EuroSCORE II and the CRS score as the lower and higher limit of a range of expected mortality rates could be an interesting approach to define the preoperative risk‐profile of a patient planned for isolated tricuspid procedures. Accordingly, we recently presented a practical and integrated decisional flow chart to guide heart team decision, surgical or interventional approach as well as patient transfer to dedicated centers.^22^ Moreover, according to the data here presented, the CRS score maybe an interesting tool to speculate late survival and might help to define the best treatment option when life expectancy is limited.

Recently, the new TRI‐SCORE has been introduced by Prof. Dreyfus. It represents a modern and interesting tool for the evaluation of TR patients.^23^ The score aimed to deal with all specific factors of tricuspid disease that were not previously considered neither in the EuroSCORE nor in the CRS Score, as right ventricular function, sing of right decompensation, dose of furosemide needed, bilirubin values. The score provides the expected in hospital mortality and should be further externally validated.

## 5 STUDY LIMITATIONS

Data collection was retrospective and the risk calculation was strictly dependent on the information present in the medical documentation. The study involved several etiologies determining TR and several patient's profile included endocarditis. Therefore differences in a patient's profile may alter the results. Several aspects may have played a special role as the decision‐making process to perform repair or replacement and the center's expertise. Distribution of cases in the different centers have been previously reported and may represent a selection bias^24^ A prospective assessment of this rare cohort of patients is the main objective of the SUR‐TRI registry data collection and is ongoing. More, the number of patients enrolled, despite already considerable for the pathology itself, is still limited and larger sample size would probably lead to statistically relevant differences in the observed trends.

## CONFLICTS OF INTEREST

The authors declare no conflicts of interest.
